# DNase-Sensitive and -Resistant Modes of Biofilm Formation by *Listeria monocytogenes*

**DOI:** 10.3389/fmicb.2015.01428

**Published:** 2015-12-22

**Authors:** Marion Zetzmann, Mira Okshevsky, Jasmin Endres, Anne Sedlag, Nelly Caccia, Marc Auchter, Mark S. Waidmann, Mickaël Desvaux, Rikke L. Meyer, Christian U. Riedel

**Affiliations:** ^1^Institute of Microbiology and Biotechnology, University of UlmUlm, Germany; ^2^Interdisciplinary Nanoscience Center and Department of Bioscience, Aarhus UniversityAarhus, Denmark; ^3^INRA, UR454 MicrobiologieSaint-Genès-Champanelle, France

**Keywords:** biofilm, *Listeria monocytogenes*, extracellular DNA, osmotic pressure, DNase

## Abstract

*Listeria monocytogenes* is able to form biofilms on various surfaces and this ability is thought to contribute to persistence in the environment and on contact surfaces in the food industry. Extracellular DNA (eDNA) is a component of the biofilm matrix of many bacterial species and was shown to play a role in biofilm establishment of *L. monocytogenes*. In the present study, the effect of DNaseI treatment on biofilm formation of *L. monocytogenes* EGD-e was investigated under static and dynamic conditions in normal or diluted complex medium at different temperatures. Biofilm formation was quantified by crystal violet staining or visualized by confocal laser scanning microscopy. Biomass of surface-attached *L. monocytogenes* varies depending on temperature and dilution of media. Interestingly, *L. monocytogenes* EGD-e forms DNase-sensitive biofilms in diluted medium whereas in full strength medium DNaseI treatment had no effect. In line with these observations, eDNA is present in the matrix of biofilms grown in diluted but not full strength medium and supernatants of biofilms grown in diluted medium contain chromosomal DNA. The DNase-sensitive phenotype could be clearly linked to reduced ionic strength in the environment since dilution of medium in PBS or saline abolished DNase sensitivity. Several other but not all species of the genus *Listeria* display DNase-sensitive and -resistant modes of biofilm formation. These results indicate that *L. monocytogenes* biofilms are DNase-sensitive especially at low ionic strength, which might favor bacterial lysis and release of chromosomal DNA. Since low nutrient concentrations with increased osmotic pressure are conditions frequently found in food processing environments, DNaseI treatment represents an option to prevent or remove *Listeria* biofilms in industrial settings.

## Introduction

*Listeria monocytogenes* (*Lm*) is a ubiquitous saprophytic soil bacterium and an opportunistic food-born human pathogen with a well characterized intracellular life-cycle (Vázquez-Boland et al., 2001; Hamon et al., 2006; Freitag et al., 2009). Severity of *Lm* infections and the symptoms of the associated disease (i.e., listeriosis) are dependent on the immune status of the patient (Hamon et al., 2006; Freitag et al., 2009). Healthy people infected with *Lm* develop only mild gastrointestinal symptoms or remain totally asymptomatic. By contrast, *Lm* may cause severe systemic infections in at-risk individuals including pregnant women, newborns, elderly people and immunocompromised patients with mortality rates of up to 30% in these groups (Hamon et al., 2006; Freitag et al., 2009). All outbreaks reported in recent years have been associated with consumption of contaminated food. In 2009–2010, a listeriosis outbreak caused by acid curd cheese was reported in Austria and Germany with a total of 34 cases, eight of which were fatal. Subsequent genotyping revealed that these cases of listeriosis were actually the result of two independent outbreaks caused by distinct strains (Rychli et al., 2014). A recent outbreak in Denmark caused by a traditional meat product has claimed 13 deaths amongst 28 cases (Ethelberg, 2014) and a nation-wide outbreak in the USA in 2011 with 147 patients and 33 deaths could be traced back to contaminated cantaloupe (McCollum et al., 2013). Since then several smaller food-related outbreaks have been recorded in the USA (http://www.cdc.gov/listeria/outbreaks/).

As a saprophytic soil organism and intracellular pathogen that causes infections via the gastrointestinal route, *Lm* is able to survive and grow under a wide range of temperatures and stressful environmental conditions including acid and osmotic stress (Milillo et al., 2012; Gahan and Hill, 2014). Inside host cells nutrients are abundantly available and temperature is at constant 37°C. By contrast, in soil and food processing environments temperature is variable, nutrients are usually scarce, and osmotic conditions are suboptimal. It is not surprising that growth of *Lm* under host-conditions differs markedly from growth under environmental conditions (Freitag et al., 2009). Important features including biofilm formation, flagellar motility, and expression of virulence genes are subject to complex regulation by several mechanisms that depend on temperature, PrfA and σB (Johansson et al., 2002; Kamp and Higgins, 2009; Toledo-Arana et al., 2009; Lemon et al., 2010; Garmyn et al., 2012). Another system involved in the switch from saprophytism to virulence is the *agr* peptide sensing system. Mutants in one of the components of the *agr* system are attenuated for virulence *in vitro* and *in vivo* (Autret et al., 2003; Riedel et al., 2009) but also show defective biofilm formation and survival in soil (Rieu et al., 2007, 2008; Riedel et al., 2009; Vivant et al., 2015).

The ability to withstand (or even grow under) harsh environmental conditions or treatments usually applied to preserve fresh and ready-to-eat food products make *Lm* a serious problem for the food industries (Valderrama and Cutter, 2013). *Lm* has been shown to form biofilms on various surfaces and in different media (Harvey et al., 2007; Di Bonaventura et al., 2008; Rieu et al., 2008; Lemon et al., 2010; Renier et al., 2011). This feature greatly facilitates survival of *Lm* in this wide spectrum of habitats and, more importantly, in food processing environments. Moreover, biofilm formation not only provides protection against harmful environmental conditions but also increases resistance to sanitizing agents (Robbins et al., 2005; Pan et al., 2006; Berrang et al., 2008).

Biofilms are single- or multispecies microbial communities, which are embedded in a self-produced matrix of extracellular polymeric substances (EPS; Hall-Stoodley et al., 2004). Depending on the microorganism (or the community), EPS is composed of proteins, polysaccharides and/or extracellular DNA (eDNA; Flemming and Wingender, 2010). eDNA was shown to be an important structural component of the EPS matrix of a wide range of Gram-positive and -negative bacteria (Okshevsky and Meyer, 2015). For *Lm*, it was shown that stationary phase cultures grown in BHI medium contained DNA (Harmsen et al., 2010). Removal of DNA from the supernatants by DNaseI treatment inhibited initial attachment of bacteria in cultures diluted in phosphate-buffered saline (PBS) to glass and markedly delayed biofilm formation of bacteria grown in minimal medium in polystyrene microtiter plates (Harmsen et al., 2010).

With the present study, the role of eDNA during biofilm formation of *Lm* was investigated at different temperatures in normal and diluted complex medium. A wide range of different media (complex and defined, full strength and diluted) and temperatures are used by different groups to study biofilm formation of *Lm* (Monk et al., 2004; Folsom et al., 2006; Pan et al., 2006; Lemon et al., 2007; Riedel et al., 2009; Harmsen et al., 2010; Garmyn et al., 2011; Guilbaud et al., 2015). For the sake of simplicity, conditions were selected that represent normal and reduced nutrient concentrations with increased osmotic pressure (normal vs. diluted complex medium) as well as flagellated or non-motile bacteria (25 vs. 37°C). The results suggest that, irrespective of the temperature, *Lm* is able to form DNase-sensitive and -resistant biofilms depending on the osmotic conditions.

## Materials and Methods

### Bacterial Strains and Culture Conditions

All *Listeria* sp. strains used in this study are listed in **Table 1.** Bacteria were cultivated in brain heart infusion broth (BHI, Oxoid) or 10-fold diluted BHI (0.1BHI) at 25 or 37°C. Where indicated PBS (2.7 mM KCl, 137 mM NaCl, 1.5 mM KH_2_PO_4_, 7 mM Na_2_HPO_4_, pH 7.4) was used instead of demineralized water to prepare diluted medium for biofilm assays. Phosphate buffer and saline were prepared by omitting KCl and NaCl or KH_2_PO_4_ and Na_2_HPO_4_, respectively. To prepare an inoculum for biofilm assays, 10 ml BHI were inoculated with a single colony from a fresh agar plate and incubated aerobically at 37°C over night (o/N).

**Table 1:** Bacterial strains used in the present study.

Species/Strain	Lineage	Serotype	Reference/Source
*L. monocytogenes* EGD-e	II	1/2a	Bécavin et al., 2014^a^
*L. monocytogenes* LO28	II	1/2c	^a^
*L. monocytogenes* 10403S	II	1/2a	Bécavin et al., 2014^a^
*L. monocytogenes* F2365	I	4b	Nelson et al., 2004^a^
*L. monocytogenes* 33032	I	1/2b	Ducey et al., 2007^a^
*L. innocua* CIP10775	–	–	^b^
*L. ivanovii* CIP78.42	–	–	^b^
*L. grayi* CIP68.18	–	–	^b^
*L. seeligeri* SLCC3954	–	–	Steinweg et al., 2010^b^


*^a^Strains were kindly provided by Pat Casey and Colin Hill, University College Cork, Ireland. ^b^Strains were kindly provided by Frederic Borges, Université de Lorraine, France.*

### Microtiter Plate Biofilm Assays

Static biofilm assays were performed using a standard microtiter plate assay as described previously (Riedel et al., 2009). An o/N culture was diluted to an optical density (OD_600_) of 0.05 in fresh BHI or 0.1BHI medium. Aliquots of 200 μl were distributed in polystyrene 96-well plates (Sarstedt) with four technical replicates per strain and condition. Where indicated, 1 unit (U) of DNaseI (Thermo Scientific) was added to the wells directly after inoculation. Plates were incubated at 25 or 37°C for 24 h. For analysis, biofilms were washed gently twice with PBS followed by staining with 0.1% (v/v) crystal violet solution (Merck) for 30 min. After three further washings with PBS crystal violet was released from biofilms by addition of 100 μl 96% (v/v) ethanol and incubated for 10 min. Biofilm biomass was quantified by measuring absorption at 562 nm (Abs_562__nm_) with background correction, i.e., crystal violet staining in wells incubated with sterile media under the same conditions. Background levels were Abs_562__nm_ = 0.10 ± 0.02 depending on the medium. In all cases, stained biomass of untreated biofilms was at least twofold above background.

### Preparation and Detection of DNA in Biofilm Supernatants

For isolation of DNA, biofilms were prepared as described above. Supernatants from at least 12 wells per sample were collected and sterilized with 0.22 μm filters (Sarstedt). Sodium chloride was added to 1 ml supernatant to a final concentration of 250 mM. DNA was precipitated with 2.5 volumes of 96% (v/v) ethanol at -20°C o/N and harvested by centrifugation. DNA was washed once with 70% (v/v) ethanol, air-dried, and then dissolved in 50 μl demineralized water. To confirm the source of the isolated DNA, PCR was performed on the following genes: prfA, secA, lmo0849 and lmo1215. The primers used are listed in **Supplementary Table S1**. Taq polymerase S (Genaxxon BioScience GmbH) was used for amplification and annealing temperatures and extension times were optimized for each amplicon/primer pair. *Lm* EGD-e chromosomal DNA was used as control. DNA was analyzed by electrophoresis on 0.8% agarose gels in 1x TAE buffer and 1 kb or 50 bp ladders (Fermentas) were used as markers.

### Analysis of Biofilms Grown Under Flow Conditions

For flow chamber biofilms, an o/N culture was diluted in fresh BHI or 0.1BHI medium to an OD_600_ of 0.05 and 200 μl of this inoculum was injected into the chamber of an IBIDI^®^ μ-slide VI^0.4^ Uncoated, which had previously been flushed with media. This inoculum was incubated for 1 h without flow in a horizontal position to allow for initial attachment of bacteria to the surface. The chamber was moved to a vertical position and flow of medium was started at a rate of 3.3 ml/h. Biofilms were incubated for 24 h at either 25 or 37°C prior to imaging. For DNaseI treatments, medium flow was turned off. Channels containing biofilms to be treated were flooded with 250 μl of a 100 μg/ml of DNaseI (247 Keunitz units/ml, Sigma) solution in PBS and incubated without flow for 1 h at room temperature prior to imaging.

### Confocal Microscopy of Biofilms

Biofilms were grown under the conditions described above. For static biofilms ibidi^®^ μ-Plate 96 Well Uncoated plates were used instead of polystyrene microtiter plates. After 24 h, medium was removed gently by aspiration, and biofilms washed three times with PBS. Biofilms were stained as described previously (Okshevsky and Meyer, 2014) in PBS containing 10 μM Syto 60^®^ (Thermo Scientific), a red-fluorescent, membrane permeable dye staining live bacteria and 2 μM TOTO-1^®^ (Thermo Scientific), i.e., a green-fluorescent dye staining eDNA or DNA of bacteria with a compromised membrane. Imaging was performed on a Zeiss LSM700 confocal laser scanning microscope (CLSM) equipped with 555 and 635 nm lasers and a variable dichroic beam splitter for simultaneous recording of the emitted light from the two fluorophores by separate photomultipliers. All images were captured with a 63× objective and analyzed using Zen 2012 software (Zeiss).

### Statistical Analysis

All experiments were performed with at least three independent bacterial cultures (biological replicates). Normal distribution of the sample populations was assumed. Data was analyzed using Student’s *t*-test or ANOVA with Bonferroni post-test analysis as indicated in the figure legends and *p* < 0.05 was considered statistically significant.

## Results

### DNaseI-Sensitive and -Resistant Modes of Biofilm Formation by *Lm*

**FIGURE 1:**
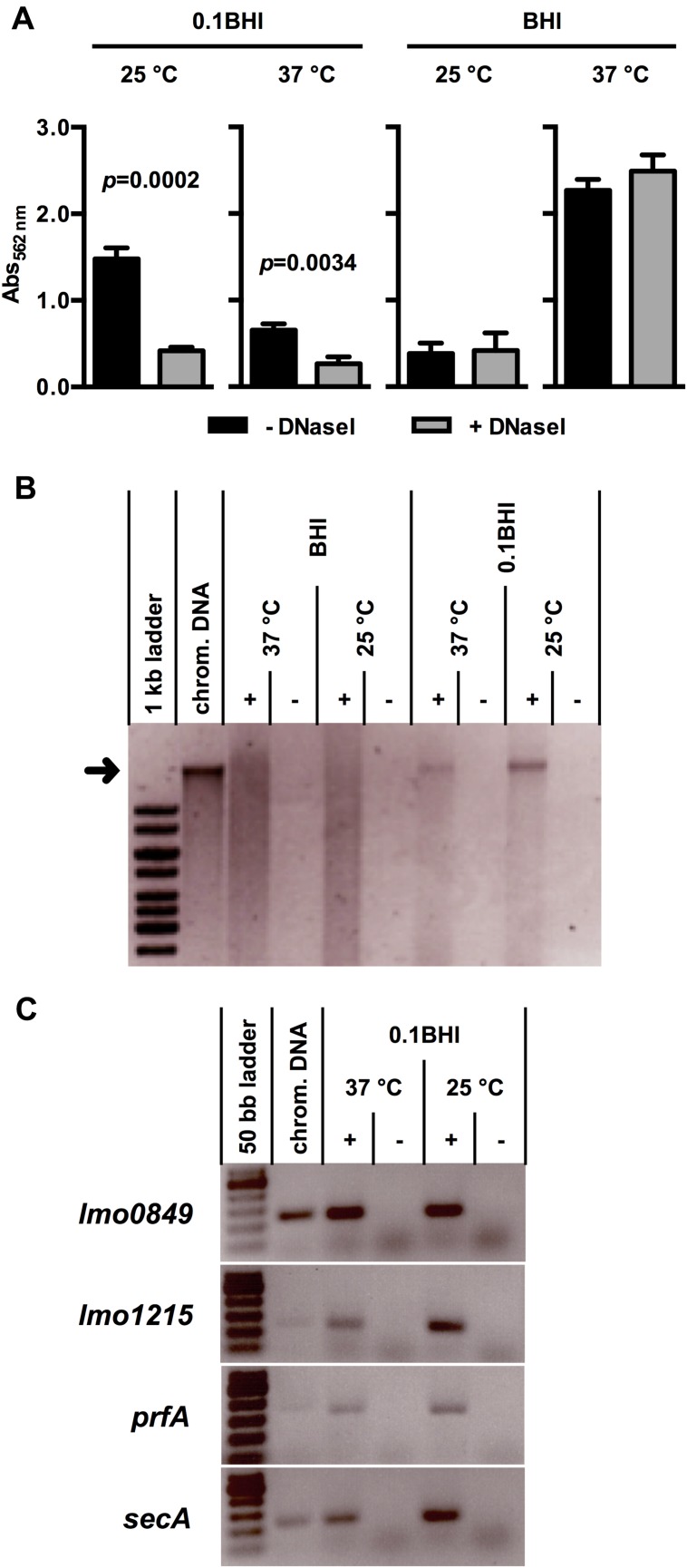
**(A)** Biofilm formation of *Lm* EGD-e grown at 37 or 25°C in BHI or 0.1BHI in the presence (gray bars) or absence (black bars) of DNaseI. Values are mean ± standard deviation of three independent experiments. Data was analyzed using Student’s *t*-test and *p*-values of statistically significant differences are indicated (all other comparisons: not significant, i.e., *p* > 0.05). **(B)** Precipitated DNA in supernatants of biofilms (+) shown in **(A)** (only biofilms without DNaseI) resolved by electrophoresis on a 0.8% agarose gel (size marker: 1 kb ladder). As controls, sterile media (-) were incubated under the same conditions and used for DNA precipitation. The size of isolated *Lm* EGD-e chromosomal DNA, which was run in a separate slot of the gel as control, is indicated by a black arrow. **(C)** PCR products targeting four genes encoded on the *Lm* EGD-e chromosome resolved by electrophoresis on a 2.0% agarose gel (size marker: 50 bp ladder). As template, DNA precipitated from biofilm grown in 0.1 BHI at 25 or 37°C (+) or sterile media controls (-) was used. *Lm* EGD-e chromosomal DNA served as a positive control for PCR reactions. In **(B)** and **(C)**, results of one representative of three independent experiments are shown.

Initial attachment of *Lm* to glass and plastic surfaces was shown previously to be dependent on eDNA and later stages of biofilm formation are sensitive to DNaseI treatment (Harmsen et al., 2010). To characterize the role of eDNA in biofilm formation of *Lm* in more detail, biofilm assays were performed in polystyrene microtiter plates under static conditions at different temperatures in full strength or 0.1BHI (**Figure 1A**). These conditions were selected to represent normal and reduced nutrient concentrations with increased osmotic pressure (normal vs. diluted complex medium) as well as flagellated or non-motile bacteria (25 vs. 37°C). Moreover, BHI and these temperatures were used in previous studies on transcriptional profiling of *Lm* EGDe (Riedel et al., 2009; Garmyn et al., 2012). After 24 h, a maximum of biomass in *Lm* biofilms was obtained in BHI at 37°C (A_562__nm_ = 2.27 ± 0.13) and lowest levels of biofilm formation were observed in the same medium at 25°C (A_562__nm_ = 0.38 ± 0.12). In 0.1BHI, biofilm biomass was higher at 25°C (A_562__nm_ = 1.48 ± 0.13) compared to 37°C (A_562__nm_ = 0.65 ± 0.07).

All experiments were performed in the presence and absence of DNaseI (**Figure 1A**). Interestingly, presence of DNaseI inhibited biofilm formation only in 0.1BHI at both temperatures and this effect was more pronounced at 25°C (37 °C: *p* = 0.0034; 25°C: *p* = 0.0002). Similar results were obtained when biofilms were grown for up to 48 h in the presence and absence of DNaseI (**Supplementary Figure S1**) or at 20 and 30°C (data not shown). Under all conditions tested, biofilms grown in 0.1BHI were sensitive to DNaseI but no significant effects of DNaseI treatment were observed in full strength BHI medium. Likewise, treatment of established biofilms for 1 h with DNaseI reduced biofilm biomass in diluted but not full strength medium and heat-inactivated DNaseI had no effect (**Supplementary Figure S2**). This demonstrates that enzymatic activity rather than presence of the protein is responsible for the observed effect.

### Presence of eDNA in *Lm* Biofilms Grown Under Static Conditions

To further investigate presence and source of eDNA, nucleic acids were precipitated from biofilm supernatants. This yielded a distinct band of high molecular weight DNA in supernatants of *Lm* biofilms grown in 0.1BHI but not in full strength medium, which corresponded to the size of isolated chromosomal DNA of *Lm* EGD-e (**Figure 1B**). To further confirm the chromosomal origin of this eDNA, PCR targeting four distinct loci randomly distributed across the *Lm* EGD-e chromosome was performed using DNA isolated from biofilm supernatants as template. For all target genes, specific products were obtained from cultures grown in 0.1BHI (**Figure 1C**) suggesting that the observed bands (**Figure 1B**) are indeed chromosomal DNA.

**FIGURE 2:**
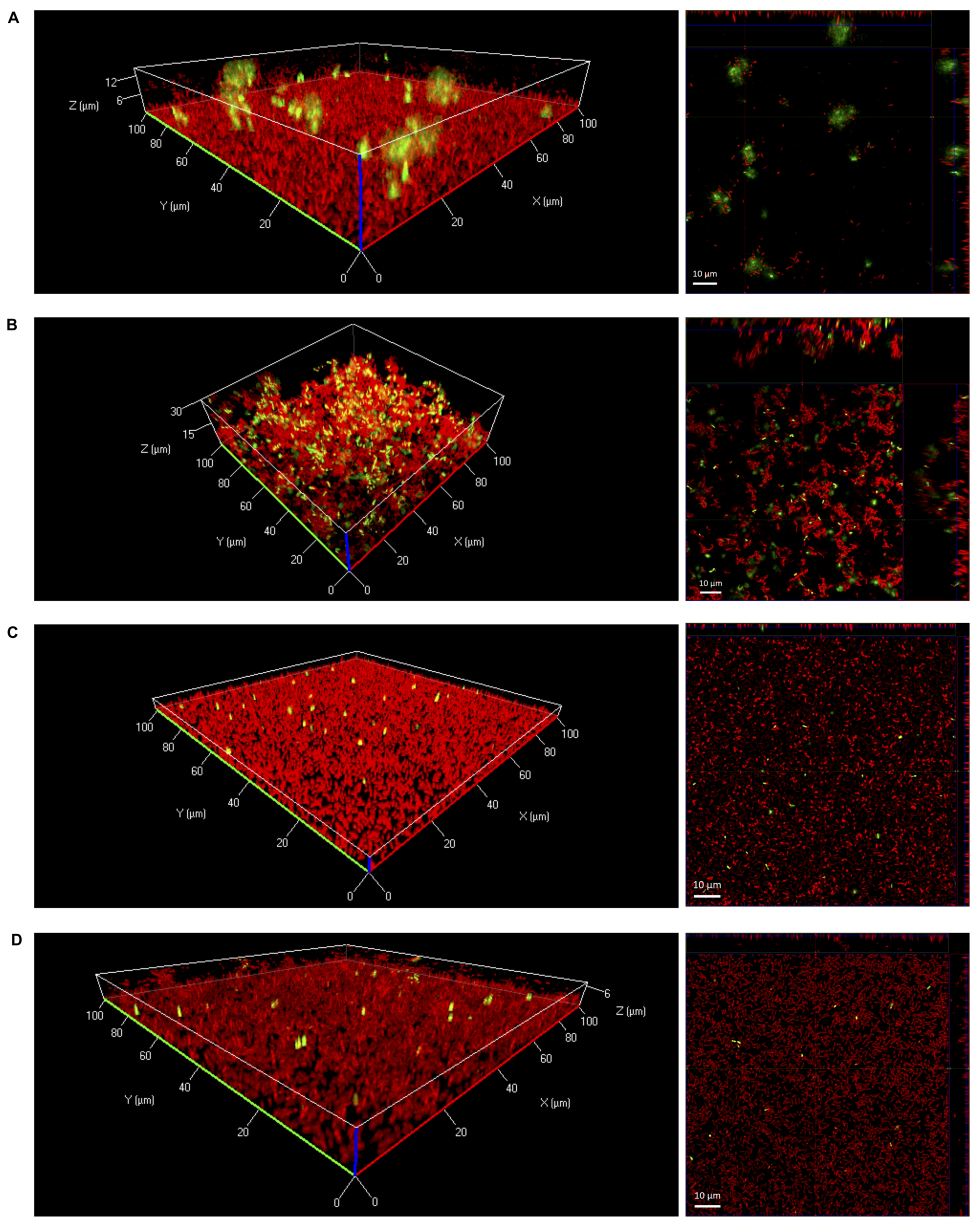
**Three-dimensional (left panels) or orthogonal projections (right panels) of CLSM Z-stack images of *Lm* EGD-e biofilms grown for 24 h under static conditions in 96-well microtiter plates.** Media and temperatures were: **(A)** 0.1BHI at 25 °C, **(B)** 0.1BHI at 37°C, **(C)** BHI at 25°C, and **(D)** BHI at 37°C. Live bacteria are stained by SYTO-60 (red) and eDNA with TOTO-1 (green). Size bars in orthogonal projections indicate 10 μm.

In further experiments, eDNA in biofilms was visualized by confocal laser scanning microscopy. After 24 h of growth under static conditions large diffuse patches of eDNA were only observed when biofilms were grown in 0.1BHI (**Figures 2A,B**). Biofilms grown in 0.1BHI had a clear three-dimensional architecture with a confluent layer of bacteria at the bottom and large, cloud-like patches of eDNA extending up to 30 μm toward the top of the biofilm (**Figures 2A,B**). In biofilms grown in 0.1BHI at 25°C, a number of bacteria appeared to be in close proximity of these eDNA clouds suggesting they might be attached to these structures. Moreover, biofilms grown in 0.1BHI at 37°C had a more complex structure with hollow domes and channels in which eDNA appeared to serve as a structural component (**Figure 2B**).

In full strength BHI, biofilms were mostly flat and rather featureless (**Figures 2C,D**). In these biofilms, only a few, well defined spots stained positive for DNA. These signals had approximately the size of SYTO-60 positive live bacteria and thus probably represent intracellular DNA of intact, dead cells with a compromised membrane rather than eDNA from lysed bacteria.

### Presence of eDNA in 0.1BHI Depends on Osmotic Conditions

**FIGURE 3:**
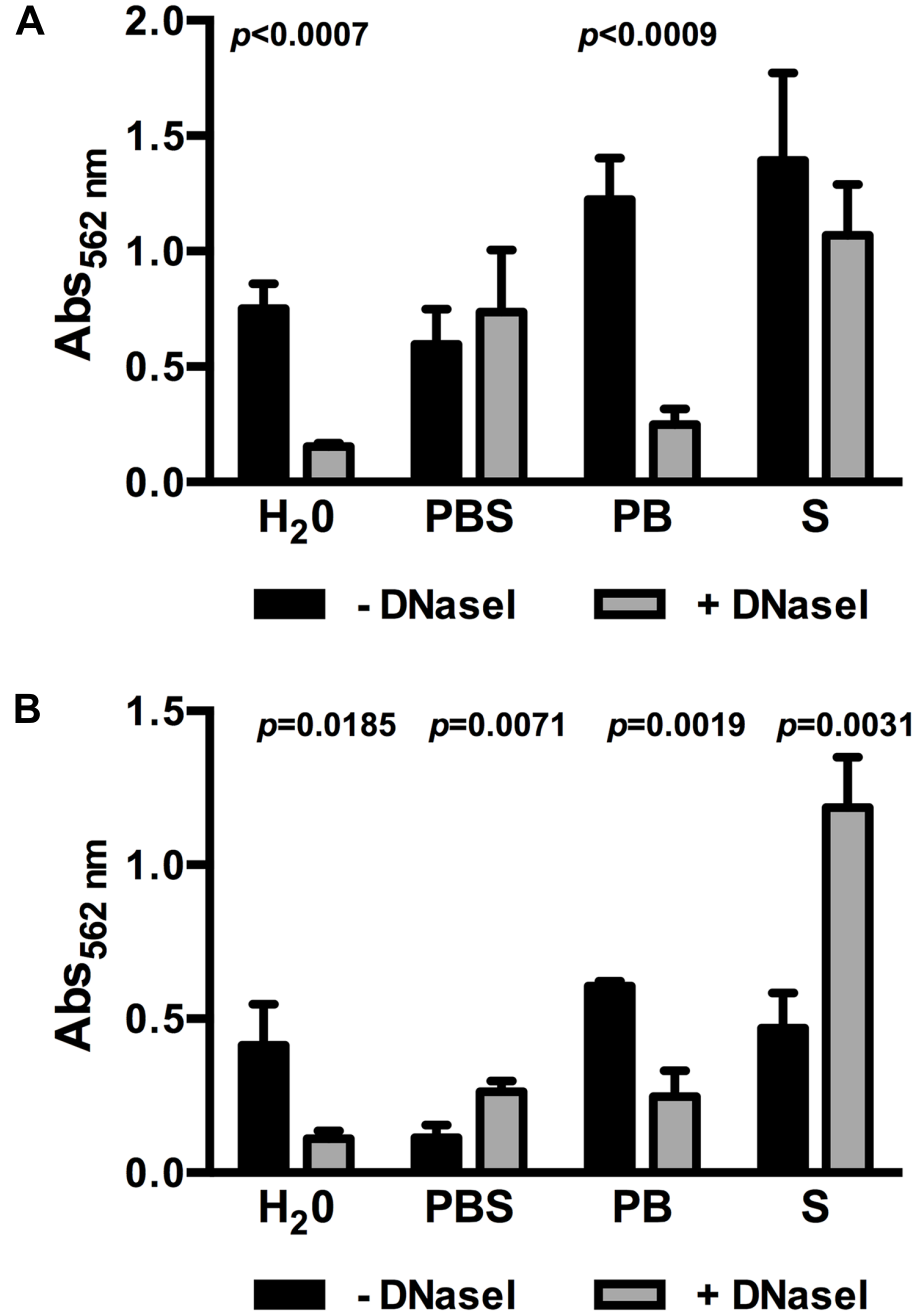
**Biofilm formation of *Lm* EGD-e grown in 0.1BHI prepared with either dH_2_O, PBS, phosphate buffer (PB), or saline (S) grown at 25°C **(A)** or 37°C **(B)** in the presence (gray bars) or absence (black bars) of DNaseI.** Values are absorbance at 562 nm and are mean ± standard deviation of three independent experiments. Data was analyzed using Student’s *t*-test and *p*-values of statistically significant differences are indicated (all other comparisons: not significant, i.e., *p* > 0.05).

One factor influencing bacterial lysis is ionic strength of the extracellular environment and, in consequence, intracellular osmotic pressure. Dilution of BHI in demineralized H_2_O to obtain 0.1BHI results in a hypotonic solution increasing the osmotic pressure. Thus, further experiments were performed to test if an increase in osmotic pressure in 0.1BHI contributes to DNase sensitivity of biofilms. At 25°C, the use of PBS to dilute BHI instead of demineralized H_2_O completely abolished the effect of DNase treatment on biofilm formation (**Figure 3A**). This effect could be attributed to the presence of higher ionic strength in PBS since a similar inhibition of DNase sensitivity was observed with saline but not phosphate buffer (**Figure 3A**). Similar observations were made at 37°C (**Figure 3B**). Again, biofilm formation was reduced by DNaseI treatment in 0.1BHI diluted with H_2_O or phosphate buffer but not with PBS or saline. Instead, addition of DNase enhanced biofilm formation in BHI diluted with PBS or saline at 37°C. To exclude any effects on enzymatic activity of DNaseI, control experiments were performed. Under all conditions tested DNaseI retained full activity (**Supplementary Figure S3**).

### Presence of eDNA in *Lm* Biofilms Grown Under Flow

**FIGURE 4:**
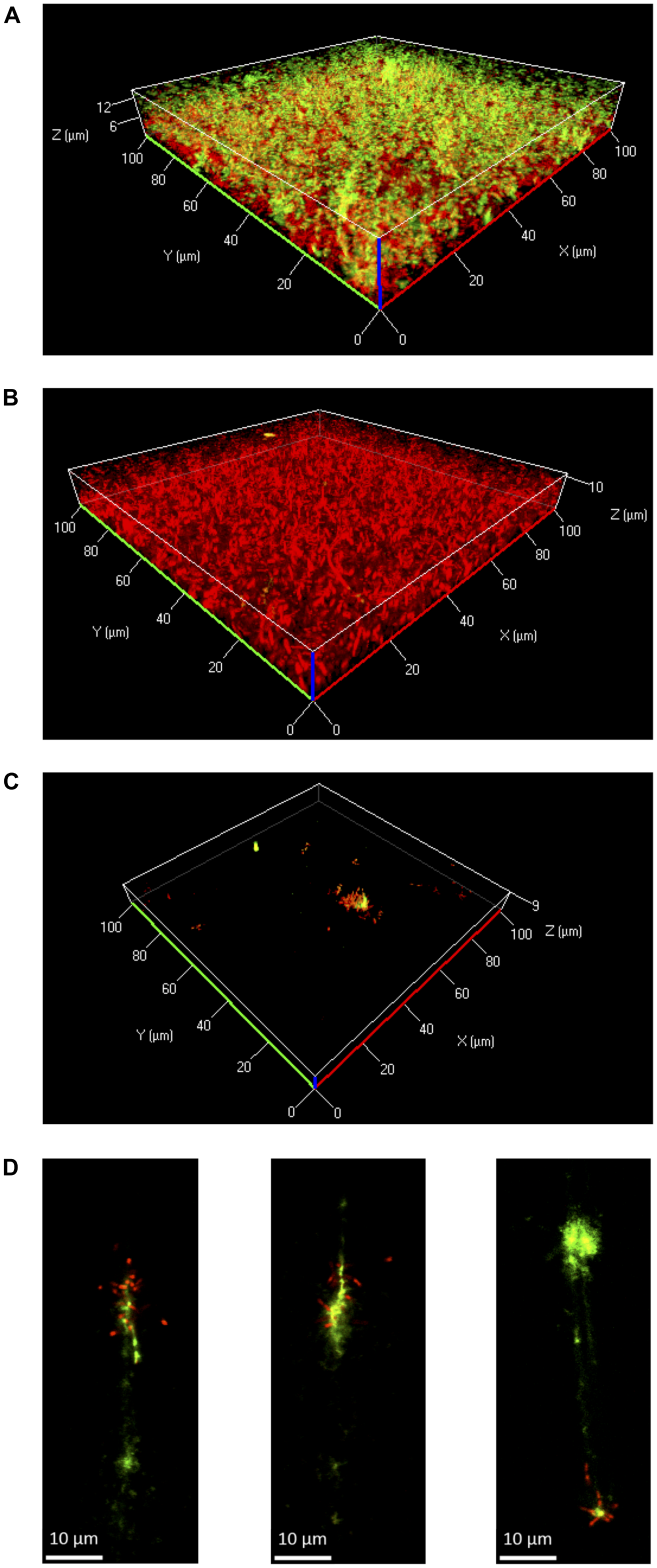
**Three-dimensional projections or single layers of CLSM images of *Lm* EGD-e flow chamber biofilms grown for 24 h under hydrodynamic conditions at 37°C in 0.1BHI **(A)** or BHI **(B)** or at 25°C in 0.1BHI **(C)** and **(D)**.** Live bacteria are stained by SYTO-60 (red) and eDNA with TOTO-1 (green). **(D)** Digital magnification of eDNA spots identified in the layer of the z-stacks that corresponds to the surface of the slide (size bar indicates 10 μm).

Further experiments were performed in flow chambers to investigate the role of eDNA in *Lm* biofilms under dynamic conditions. Confocal microscopy analysis of eDNA in biofilms grown in full strength and diluted BHI at 25 or 37°C revealed a similar picture as in static biofilm assays. At 37°C, large amounts of eDNA were present in biofilms grown in 0.1BHI and appeared to be a structural component of the matrix throughout the entire biofilm from the bottom to the top (**Figure 4A**; **Supplementary Figure S4**). By contrast, only very few dead cells or small patches of eDNA were present in biofilms grown in full strength BHI (**Figure 4B**; **Supplementary Figure S4**).

At 25°C, flow chamber biofilms differed considerably compared to those formed under static conditions. Under flow, only few isolated microcolonies were observed in 0.1BHI and these microcolonies were mostly found around patches of eDNA (**Figure 4C**; **Supplementary Figure S4**). Upon higher magnification, the eDNA patches appeared as filamentous structures directly on the slide surface, which had several bacteria attached (**Figure 4D**). In full strength BHI, only a few isolated bacteria were found to be attached to the surface and no eDNA, microcolonies, or biofilm were observed (**Supplementary Figure S4**).

**FIGURE 5:**
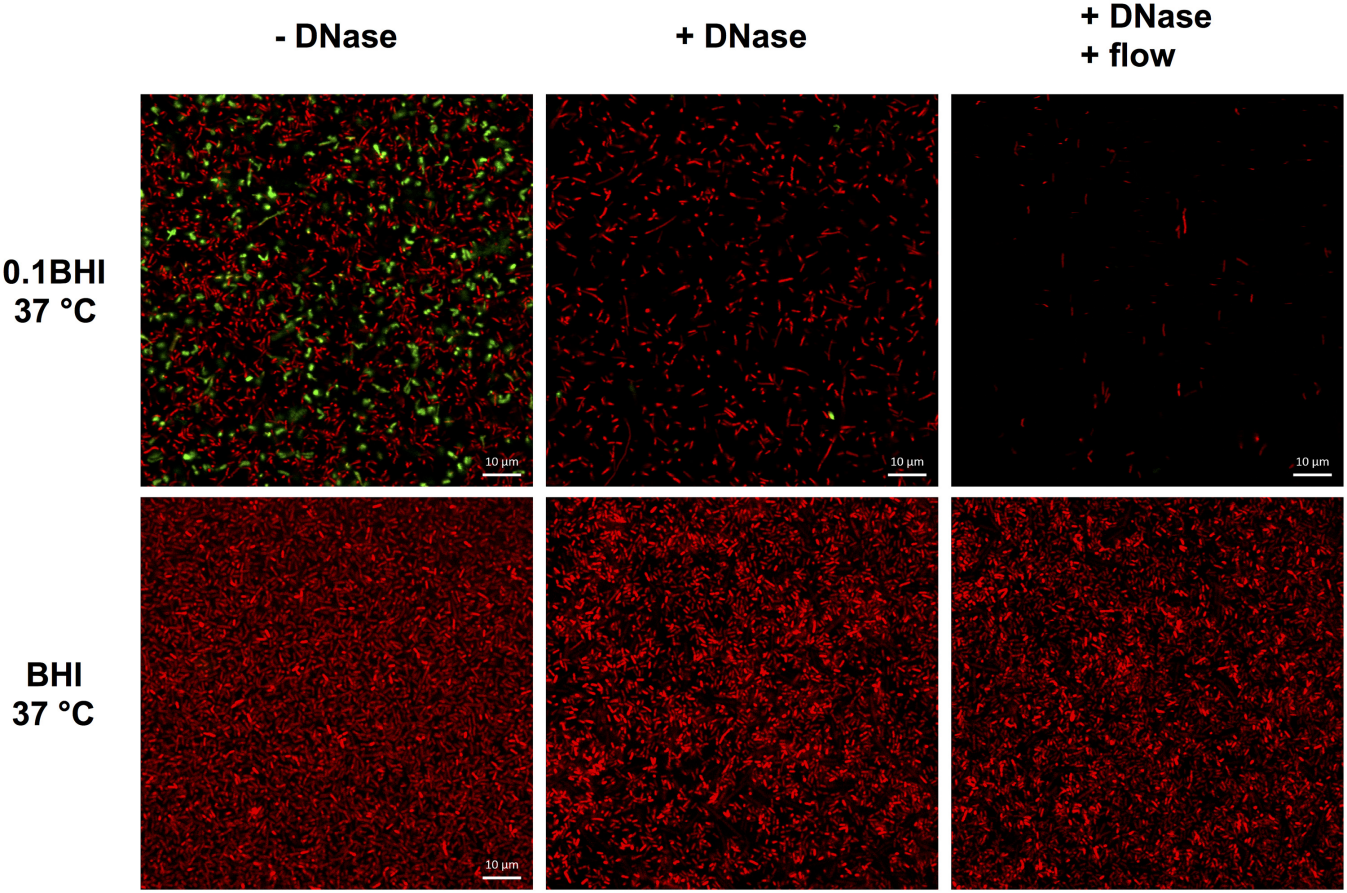
**Confocal laser scanning microscope (CLSM) images of *Lm* EGD-e flow chamber biofilms grown for 24 h under hydrodynamic conditions at 37°C in 0.1BHI (upper panels) or BHI (lower panels).** Images were captured at the basal layer of bacteria immediately above the slide surface and at the same position before (left, -DNase) and at the end of DNaseI treatment (middle, +DNase) and after medium flow had been turned on again (right, +DNase + flow). Live bacteria are stained by SYTO-60 (red) and eDNA with TOTO-1 (green). Size bars indicate 10 μm.

Based on these results, the potential of DNaseI-treatment to dissolve established biofilms of *Lm* was investigated. After 1 h of incubation, eDNA in biofilms grown in 0.1BHI was efficiently digested by DNaseI (**Figure 5**; **Supplementary Figure S5**). Moreover, these biofilms were almost completely removed after flow was turned on again. By contrast, biofilms grown in full strength medium were unaffected by DNaseI treatment probably due to the lack of eDNA (**Figure 5**).

### DNase-Sensitive and -Resistant Biofilms of Different *Listeria* sp. Strains

**FIGURE 6:**
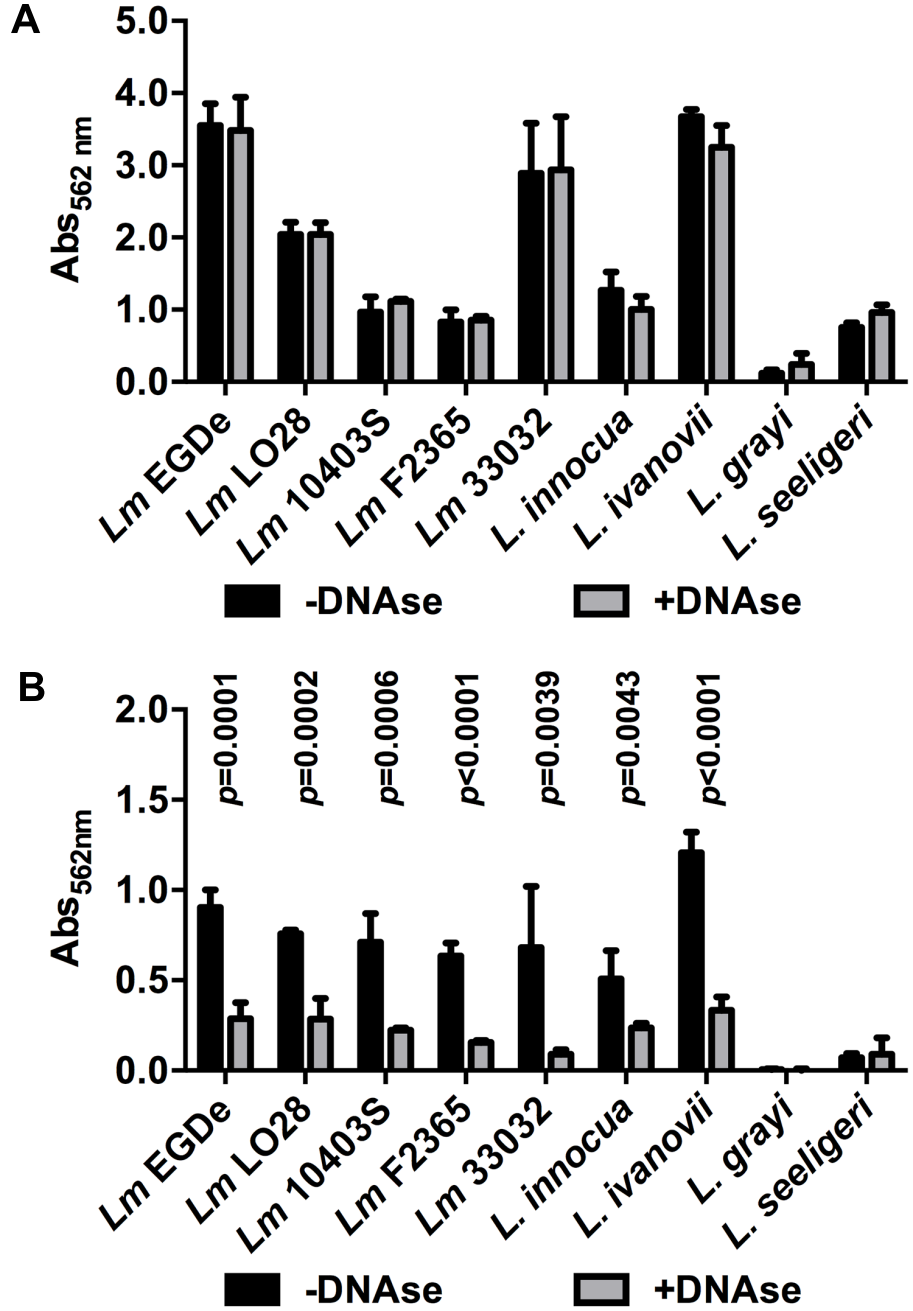
**Biofilm formation of different *Lm* strains and other *Listeria* species grown in BHI at 37°C **(A)** or 0.1BHI at 25°C **(B)** in the presence (gray bars) or absence (black bars) of DNaseI.** Values are mean ± standard deviation of three independent experiments. Data was analyzed using Student’s *t*-test and *p*-values of statistically significant differences are indicated (all other comparisons: not significant, i.e., *p* > 0.05).

Finally, a range of *Lm* strains from different lineages as well as different species of the genus *Listeria* were tested for DNase-sensitive and -insensitive modes of biofilm formation under static conditions. All *Lm* strains as well as *L. innocua* and *L. ivanovii* formed DNase-insensitive biofilms at 37°C in full strength BHI (**Figure 6A**) but biofilm formation was reduced by DNaseI in 0.1BHI at 25°C (**Figure 6B**). Similar DNase-sensitive and in-sensitive biofilms were observed for these strains grown in 0.1BHI at 37°C or BHI at 25°C (data not shown). No significant biofilm formation was observed for *L. grayi* under all conditions tested and *L. seeligeri* only formed DNase-resistant biofilms in BHI at 37°C.

## Discussion

### Biofilm Formation Depends on Temperature and Dilution of Media

A number of studies have investigated the impact of nutrients and temperature on biofilm formation of various *Lm* strains (Folsom et al., 2006; Harvey et al., 2007; Di Bonaventura et al., 2008). The media and conditions vary from study to study but the authors uniformly report a strain-specific pattern with some strains forming more biofilm in full strength complex media while others form more biofilm in diluted or chemically defined media. In line with previous findings (Riedel et al., 2009), the ability of *Lm* EGD-e to form biofilms varies with temperature and dilution of media. Highest levels of biofilm formation by *Lm* EGD-e were achieved at 37°C in full strength BHI, i.e., high nutrient levels, and least biofilm formation was observed at 25°C in the same medium. By contrast, in 0.1BHI biofilm formation was increased at 25°C compared to 37°C.

### eDNA-Dependent and -Independent Biofilm Formation

The importance of eDNA during early phases of biofilm formation has been established for a number of bacteria (Whitchurch et al., 2002; Qin et al., 2007; Gödeke et al., 2011; Barnes et al., 2012). The results of the present study confirm a role of eDNA for biofilm formation in media with low concentrations of osmotically active substances (**Figure 1**). Similar observations were made in a previous study showing that eDNA is important for initial attachment of *Lm* EGD-e, to glass (Harmsen et al., 2010). Moreover, the authors report inhibition of biofilm formation in minimal medium and removal of biofilms established in diluted BHI by DNaseI. In another study, presence of DNaseI markedly reduced biofilm formation on polystyrene of three *Lm* strains including EGD-e in full strength TSB medium at 37°C (Nguyen and Burrows, 2014). By contrast, biofilm formation in full strength BHI was not affected by DNaseI, suggesting that, under these conditions, eDNA is neither involved in initial attachment nor during later stages of biofilm formation.

A smear of nucleic acids could be precipitated from supernatants of biofilm grown under all conditions. This signal was by far more prominent in full strength medium and an additional distinct band was only observed in diluted medium (**Figure 1B**). This nucleic acid is clearly of bacterial origin since it is absent in sterile media. Control PCRs yielded specific products for all genes tested in supernatants of bacteria grown in diluted medium. For some of the genes tested, PCR products were also obtained when PCR was performed on supernatants of full strength BHI cultures at 25°C although the band signals were very faint at the same number of PCR cycles (**Supplementary Figure S6**) suggesting that the amount of template DNA was significantly lower compared to 0.1BHI supernatants. This indicates that the DNA smear in full strength BHI represents fragmented chromosomal DNA, which is not functional in promoting biofilm formation. Similar observations were made by Harmsen et al. (2010), who could show that, unlike intact chromosomal DNA, shorter DNA fragments do not support initial attachment of *Lm*.

So far, DNA-dependent and -independent modes of biofilm formation have only been described in *Neisseria meningitidis* (Lappann et al., 2010). However, in this organism the two modes of biofilm formation were not shown for the same strains but are distributed amongst different clonal complexes. Pathogenic strains of clonal complexes with high prevalence form eDNA-dependent biofilms that are more resistant to shear forces, possibly leading to a more stable interaction with the host. Strains of other clonal complexes show an eDNA-independent mode of biofilm formation with less stable microcolonies. Our results suggest that, in contrast to *N. meningitidis*, *Lm* EGD-e is able to form biofilms that either contain or lack eDNA in response to different environmental conditions and eDNA promotes biofilm formation specifically under conditions with low concentrations of osmotically active substances. Moreover, DNA-dependent and -independent modes of biofilm formation seem to be conserved in the species *Lm* and was also observed in other but not all species of the genus.

### Source of eDNA in *Lm* Biofilms

Several studies have investigated the source of eDNA in bacterial biofilms. Dilution of BHI in PBS or saline but not H_2_O or phosphate buffer abolished the effect of DNaseI on biofilm formation (**Figures 3A,B**) arguing for a contribution of the osmotic conditions to DNA release. In other bacteria, eDNA was released upon expression of autolysin genes (Qin et al., 2007; Rice et al., 2007; Mann et al., 2009; Lappann et al., 2010), induction of prophages in a subpopulation of the biofilm bacteria (Carrolo et al., 2010; Gödeke et al., 2011; Petrova et al., 2011; Binnenkade et al., 2014) or formation of vesicles (Allesen-Holm et al., 2006; Liao et al., 2014). Additionally, based on the observation that some bacteria employ type IV secretion systems for conjugational gene transfer, injection of DNA into host cells and active secretion of chromosomal DNA (Hamilton et al., 2005; Alvarez-Martinez and Christie, 2009), active and lysis-independent export of DNA was proposed as another source of eDNA. Further experiments are required to investigate if these mechanisms contribute to release of eDNA by *Lm*.

### eDNA as a Structural Component of *Lm* Biofilms

Microscopic images provide evidence that eDNA not only supports initial attachment but also serves as a structural component of the biofilm matrix of *Lm* EGD-e in diluted media under both static and dynamic conditions (**Figures 2** and **4**). Extracellular DNA was shown to be present in the matrix of mature biofilms of various bacteria (Hall-Stoodley et al., 2008; Izano et al., 2008; Mann et al., 2009; Seper et al., 2011; Liao et al., 2014) cooperating with proteins and polysaccharides to ensure structural integrity of the biofilm (Das et al., 2013; Okshevsky and Meyer, 2015). As a consequence, eDNA is discussed as a target to prevent or disperse biofilm formation of these microorganisms (Okshevsky et al., 2015).

### Biofilm Formation by *Lm* Under Static vs. Dynamic Conditions

Under dynamic conditions, significant biofilm formation was only observed when bacteria were grown at 37°C but not at 25°C, i.e., when bacteria express flagella. A possible explanation is that under static conditions in microtiter plates, when bacteria are located in a confined space, motility facilitates multiple contacts with the surface eventually leading to initial attachment. In fact, flagellar motility was shown to be required for efficient biofilm formation by *Lm* under static conditions (Lemon et al., 2007). Also, under these conditions non-motile strains were shown to form less structured, more homogenous biofilms (Guilbaud et al., 2015). By contrast, in flow chambers, motility might actually have the opposite effect on biofilm formation: motile bacteria that do not attach are efficiently washed away. However, once single, attached bacteria lyse under conditions of increased osmotic pressure (i.e., in 0.1BHI), eDNA may serve as attachment site for further bacteria. This is supported by the fact that filamentous eDNA patches were observed in 0.1BHI at 25°C (**Figure 4D**). These eDNA filaments were orientated in the direction of the medium flow and are presumably chromosomal DNA released from lysed bacteria, which was then spread out by medium flow. This sticky DNA may then serve as attachment site or scavenger for further bacteria leading to formation of microcolonies observed at lower magnification (**Figure 4C**). It remains to be investigated if these microcolonies develop into mature biofilm upon longer incubation periods.

## Conclusion

Based on the presented results a hypothetical model for biofilm formation of *Lm* is proposed. 37°C and high levels of nutrients are conditions encountered by *Lm* in the gastrointestinal tract of the host. On the other hand, 25°C and low levels of nutrients and other osmotically active substances are conditions encountered in the environment and in food production lines. It may thus be hypothesized that efficient and rapid formation of DNA-independent biofilms on, e.g., food particles or host tissue contributes to colonization and prolonged persistence of *Lm* and, in consequence, to sustained exposure to the pathogen. By contrast, under environmental conditions, eDNA released by lysed bacteria (or present in the environment) supports initial attachment to surfaces. Hypotonic conditions may favor increased lysis of bacteria already attached to the surface. The chromosomal DNA released by these lysed bacteria then serves as an anchoring site for dividing cells in growing microcolonies but also a scavenger capturing further planktonic bacteria.

Collectively, the presented results may have practical implications for contact surfaces in food production lines at risk for contamination by *Lm*. Targeting eDNA in the biofilm matrix by DNases or nucleases, as suggested for other bacteria (Nguyen and Burrows, 2014; Okshevsky et al., 2015), may be an effective treatment to limit or prevent initial attachment and disperse already existing *Lm* biofilms.

## Author Contributions

MD, RM, and CR conceived the study. MZ, MO, JM, AS, NC, MA, and MW carried out experiments. MZ, MO, MD, RM, and CR analyzed data. MZ, MO, RM, and CR drafted the manuscript and all the authors contributed to preparing the final version of the manuscript. All authors read and approved the final manuscript.

## Conflict of Interest Statement

The authors declare that the research was conducted in the absence of any commercial or financial relationships that could be construed as a potential conflict of interest.
