# Supplementary material for: DNase-Sensitive and -Resistant Modes of Biofilm Formation by Listeria monocytogenes
Source: Front Microbiol. 2015 Dec 22;6:1428. doi: 10.3389/fmicb.2015.01428 (PMC4686886; doi:10.3389/fmicb.2015.01428)
Supplement: Supplementary file 1 [file Presentation_1.PDF]

Supplementary Material

eDNA-dependent and -independent modes of biofilm formation by *Listeria monocytogenes*

Marion Zetzmann[1,#], Mira Okshevsky[2,#], Jasmin Endres[1], Anne Sedlag[1], Nelly Caccia[3], Marc Auchter[1], Mark S. Waidmann[1], Mickaël Desvaux[3], Rikke L. Meyer[2], and Christian U. Riedel[1,*]

*** Correspondence:** Dr. Christian Riedel, Institute of Microbiology and Biotechnology, University of Ulm, Albert-Einstein-Allee 11, 89081 Ulm, Germany, christian.riedel@uni-ulm.de

1 Supplementary Figures and Tables

1.1 Supplementary Figures

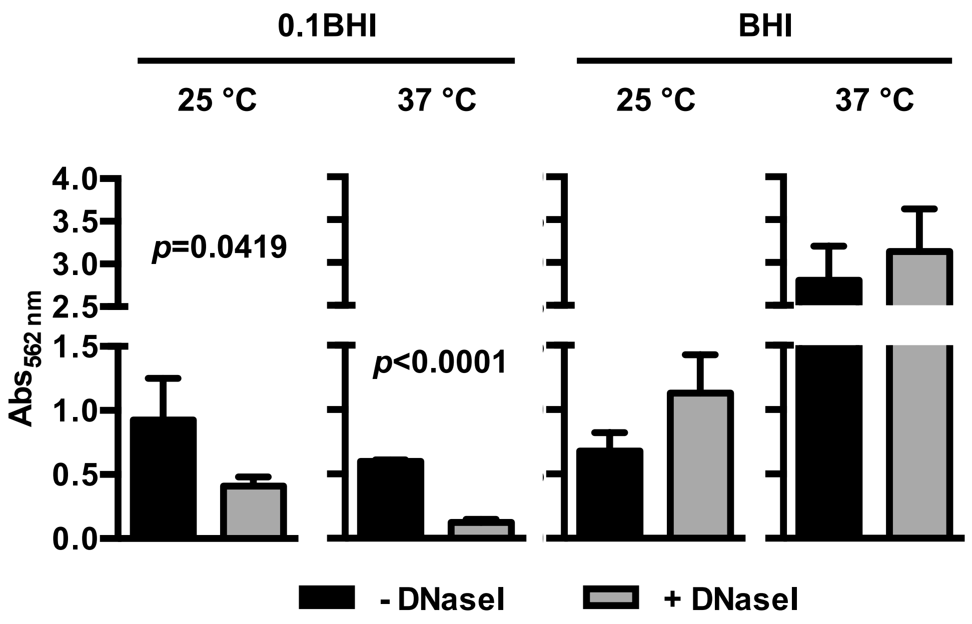

Supplementary Figure 1. Effect of DNaseI after 48 h of biofilm formation. *Lm* EGD-e was grown for 48 h at 37 °C or 25 °C in BHI or 0.1BHI in the presence (grey bars) or absence (black bars) of DNaseI. Values are mean ± standard deviation of three independent experiments. Data was analyzed using Student's *t*-test and *p*-values of statistically significant differences are indicated (all other comparisons: not significant, i.e. $p > 0.05$).

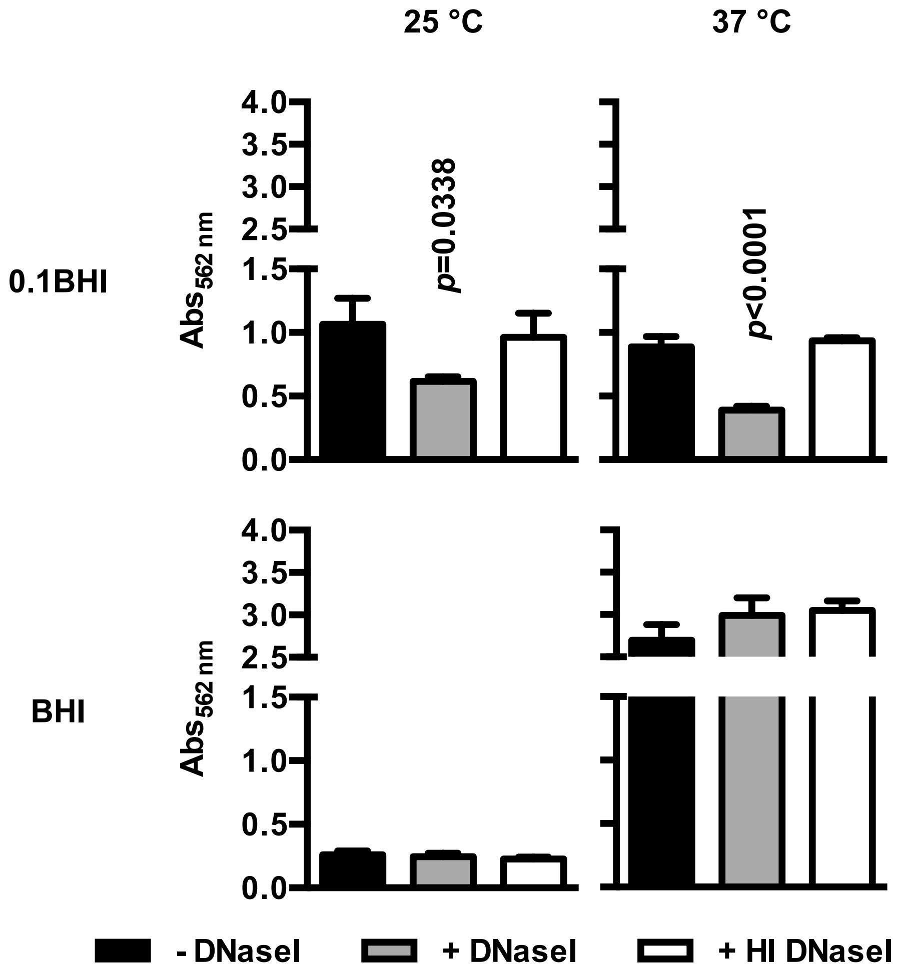

Supplementary Figure 2. Effect of native and heat-inactivated DNase in established biofilms. *Lm* EGD-e was grown for 24 h at 37 °C or 25 °C in BHI or 0.1BHI. Established biofilms were then treated with native (+ DNaseI) or heat inactivated (+ HI DNaseI) DNaseI. Biofilms treated with buffer only served as controls (- DNaseI). Values are mean ± standard deviation of three independent experiments. Data was analyzed using ANOVA with Bonferroni post-test analysis, *p*-values of statistically significant differences compared to untreated biofilms are indicated (all other comparisons: not significant, i.e. $p > 0.05$).

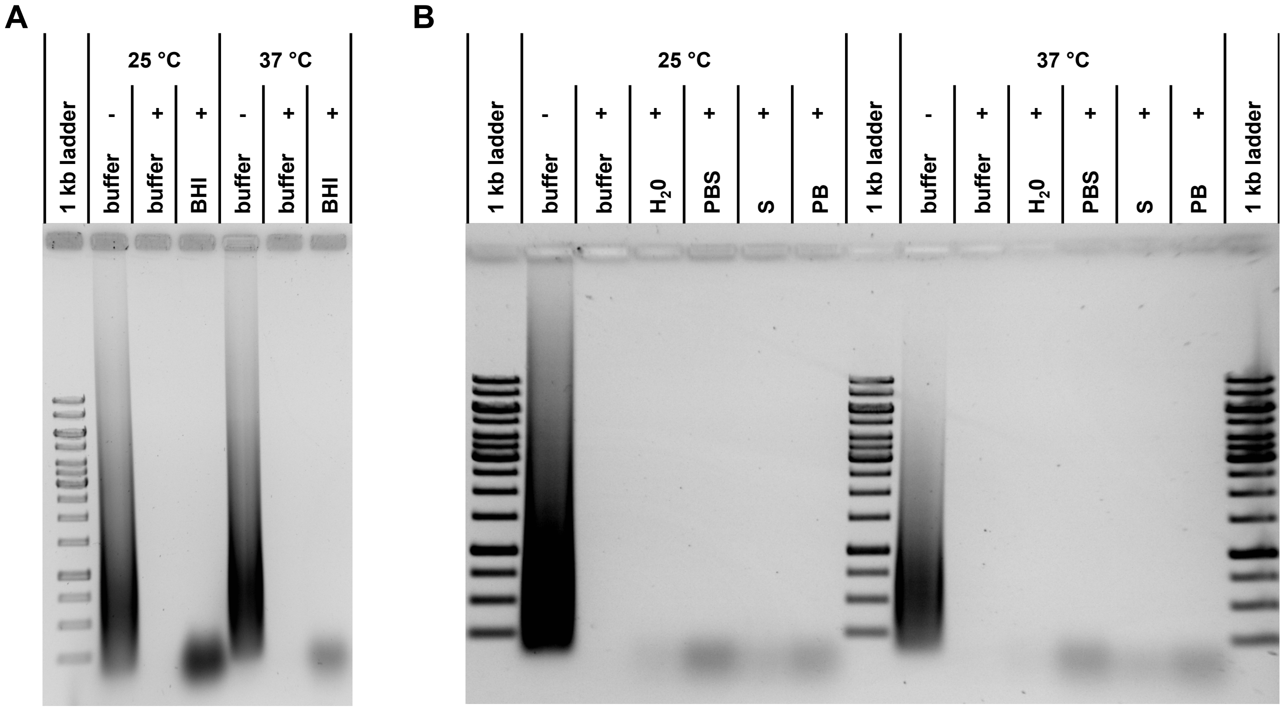

Supplementary Figure 3. DNaseI activity in full strength (A) or diluted BHI (B) at 25 or 37 °C. Hering sperm DNA was incubated with DNaseI (+) in full strength BHI or BHI diluted with H_2O, PBS, saline (S), or phosphate buffer (PB) at 25 or 37 °C for 2 h and analyzed on a 0.8 % agarose gel. As positive control, DNA was incubated under the same conditions in DNAse buffer, DNA in buffer without DNaseI (-) served as negative control. Size marker: 1kb ladder.

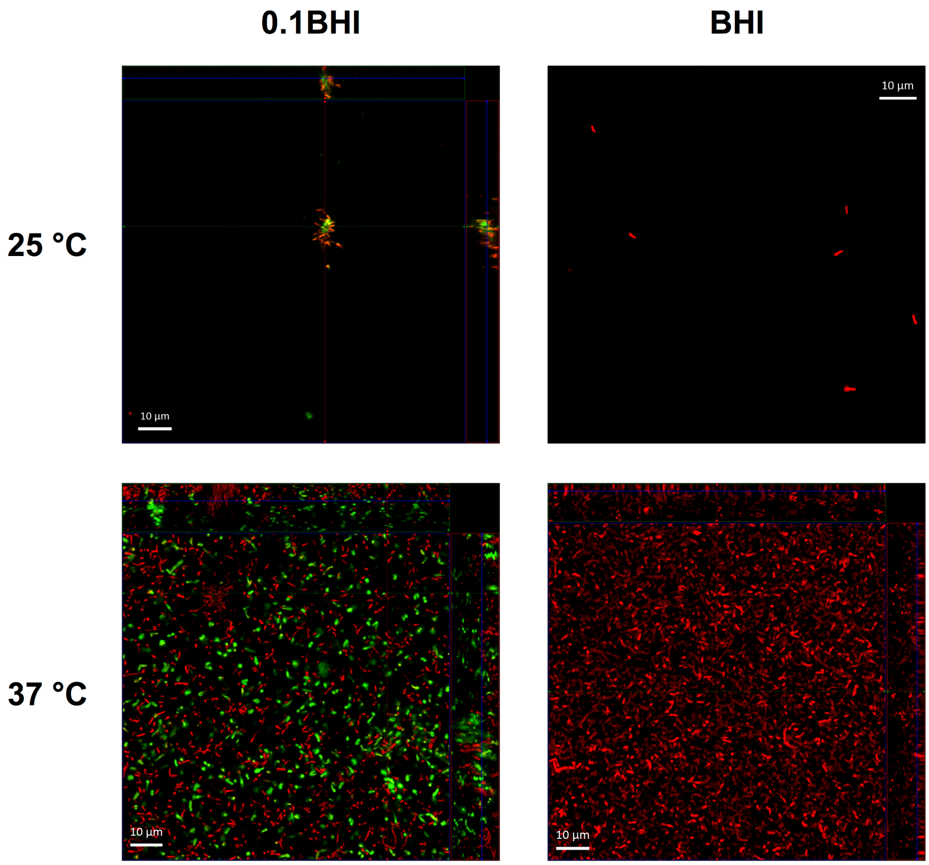

Supplementary Figure 4. Orthogonal projections of CLSM Z-stack images of *Lm* EGD-e flow chamber biofilms. *Lm* EGD-e biofilms were grown for 24 h under hydrodynamic conditions at 25 °C (upper panels) or 37 °C (lower panels) in 0.1BHI (right panels) or BHI (left panels). Live bacteria are stained by SYTO-60 (red) and eDNA with TOTO-1 (green). Size bars indicate 10 µm.

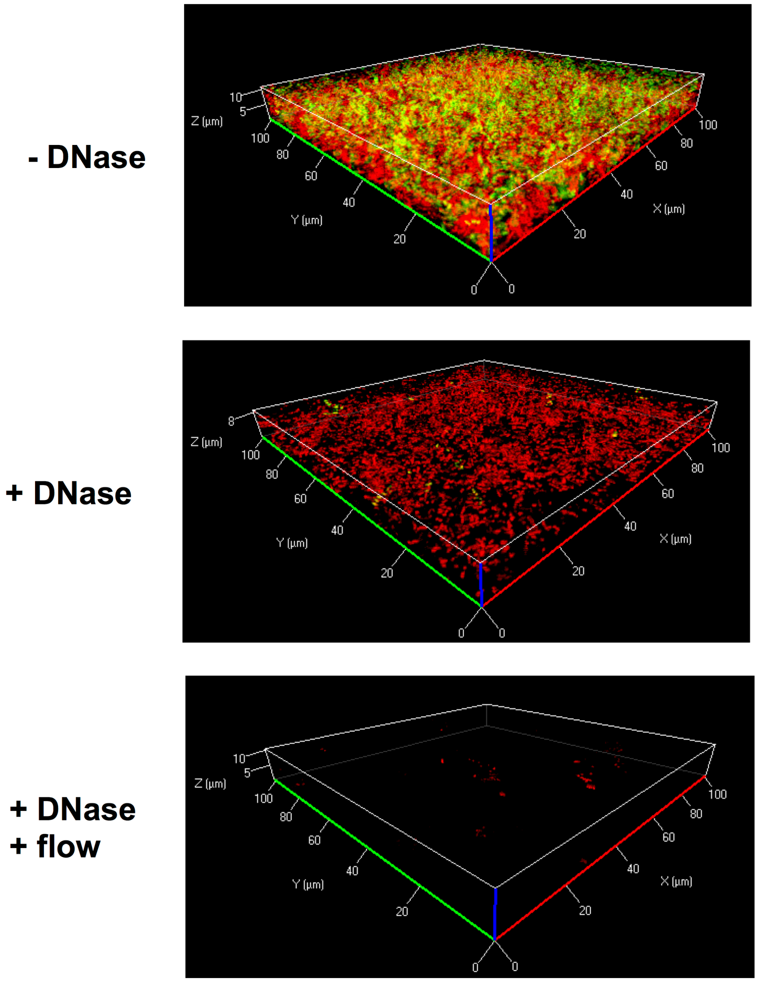

Supplementary Figure 5. Three-dimensional projections of CLSM z-stack images of *Lm* EGD-e flow chamber biofilms treated with DNaseI. *Lm* EGD-e biofilms were grown for 24 h under hydrodynamic conditions at 37 °C in 0.1BHI. Images were captured at the same position before (top, -DNase) and at the end of DNaseI treatment (middle, +DNase) and after medium flow had been turned on again (bottom, +DNase +flow). Live bacteria are stained by SYTO-60 (red) and eDNA with TOTO-1 (green).

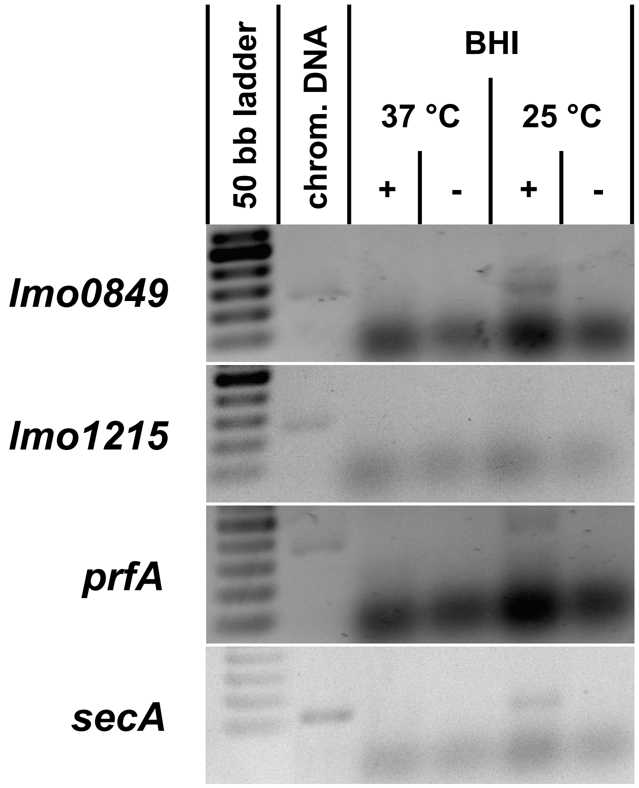

Supplementary Figure 6. PCR on biofilm supernatants grown in full strength BHI. PCR products targeting four genes encoded on the *Lm* EGD-e chromosome resolved by electrophoresis on a 2.0 % agarose gel. As template, DNA precipitated from biofilm grown in BHI at 25°C or 37°C (+) or sterile media controls (-) was used. *Lm* EGD-e chromosomal DNA served as a positive control for PCR reactions. Size marker: 50 bp ladder. Results of one representative of three independent experiments are shown.

1.2 Supplementary Tables

Supplementary Table 1: Primers used in the present study.

Primer	Sequence (5'-->3')	Target gene	Reference
prfA_f	CAGGCTACCGCATACGTTATCAAA	*prfA*	(Joseph et al., 2006)
prfA_r	AGCCAAGCTTCCCGTTAATCGAAA		
secA_f	GGCTCTTTCGGATGATGCTC	*secA*	this work
secA_r	CACCTTCTCTGGCAACAGC		
lmo0849_f	AGCTACGCTAGATTTGACTGTTC	*lmo0849*	this work
lmo0849_r	CGGCTAATTCTTTGTAGCTAAGTTG		
lmo1215_f	AACTGACCCAGATTATGCGG	*lmo1215*	this work
lmo1215_r	ACATTGCCAAAGCCAGTTG		

1.3 Supplementary References

Joseph, B., Przybilla, K., Stühler, C., Schauer, K., Slaghuis, J., Fuchs, T. M., et al. (2006). Identification of Listeria monocytogenes genes contributing to intracellular replication by expression profiling and mutant screening. *J. Bacteriol.* 188, 556–68. doi:10.1128/JB.188.2.556-568.2006.
